# Toward the next generation EGFR inhibitors: an overview of osimertinib resistance mediated by EGFR mutations in non-small cell lung cancer

**DOI:** 10.1186/s12964-023-01082-8

**Published:** 2023-04-11

**Authors:** Yufeng Li, Tianyu Mao, Jing Wang, Hongrui Zheng, Ziyi Hu, Pingping Cao, Suisui Yang, Lingyun Zhu, Shunyao Guo, Xinfei Zhao, Yue Tian, Hua Shen, Fan Lin

**Affiliations:** 1 Department of Medical Oncology, The Affiliated Sir Run Run Hospital of Nanjing Medical University, XueHai Building A111, 101 Longmian Avenue, Jiangning District, Nanjing, Jiangsu China; 2 Department of Cell Biology, School of Basic Medical Sciences, Nanjing Medical University, Nanjing, Jiangsu China; 3 Institute for Brain Tumors and Key Laboratory of Rare Metabolic Diseases, Nanjing Medical University, Nanjing, Jiangsu China; 4 Department of Gastroenterology, The First Affiliated Hospital, and College of Clinical Medicine of Henan University of Science and Technology, Luoyang, Henan China

**Keywords:** Osimertinib, Non-small cell lung cancer, Drug resistance, EGFR, Targeted therapy

## Abstract

Epidermal growth factor receptor tyrosine kinase inhibitor (EGFR-TKI) is currently the standard first-line therapy for *EGFR*-mutated advanced non-small cell lung cancer (NSCLC). The life quality and survival of this subgroup of patients were constantly improving owing to the continuous iteration and optimization of EGFR-TKI. Osimertinib, an oral, third-generation, irreversible EGFR-TKI, was initially approved for the treatment of NSCLC patients carrying *EGFR* T790M mutations, and has currently become the dominant first-line targeted therapy for most *EGFR* mutant lung cancer. Unfortunately, resistance to osimertinib inevitably develops during the treatment and therefore limits its long-term effectiveness. For both fundamental and clinical researchers, it stands for a major challenge to reveal the mechanism, and a dire need to develop novel therapeutics to overcome the resistance. In this article, we focus on the acquired resistance to osimertinib caused by *EGFR* mutations which account for approximately 1/3 of all reported resistance mechanisms. We also review the proposed therapeutic strategies for each type of mutation conferring resistance to osimertinib and give an outlook to the development of the next generation EGFR inhibitors.

Video Abstract

**Supplementary Information:**

The online version contains supplementary material available at 10.1186/s12964-023-01082-8.

## Introduction

Lung cancer is one of the leading causes of cancer death worldwide, and about 85% of lung cancer is non–small cell lung cancer (NSCLC) [1, 2]. Especially the prognosis for lung cancer is often poor because most patients have already been in the advanced stage at the time of diagnosis [3]. However, this situation has been partly changed by the in-depth understanding of molecular biology in lung cancer and the rapid advancement of drug development in the recent decade [4]. The success of targeted therapy, including monoclonal antibodies (mAbs), tyrosine kinase inhibitors (TKIs), and immunotherapy (immune checkpoint inhibitors, ICIs), has completely changed the treatment landscape of NSCLC [5–7]. By targeting key oncogenic driver gene mutations, the concept of precision medicine has been widely applied in the field of targeted therapy for lung cancer.

Among NSCLC patients, activating mutations of *EGFR* are the most common, and about 10–15% of Caucasians and 30–40% of Asian patients with non-squamous histology carry such mutations [8]. The firstgeneration EGFR-TKI represented by gefitinib and erlotinib, and the second generation EGFR-TKI represented by afatinib and dacomitinib have achieved desirable efficacy in the treatment of *EGFR*-mutant NSCLC patients [9]. However, most NSCLC patients develop drug resistance 9–14 months after the initiation of first- and second-generation targeted therapy, and the most common resistance mechanism is caused by *EGFR* exon20 T790M mutation (incidence ≥ 50%) [10]. The T790M mutation spatially hinders the binding of the first and second-generation TKIs to the ATP -binding site of EGFR, causing the inability to inhibit its activity [11]. To overcome the T790M-mediated resistance, the third-generation EGFR-TKI represented by osimertinib (Tagrisso™, trade name Teresa) emerged as the times require, which can target the T790M mutation while hardly affecting the wild-type epidermal growth factor receptor (WT EGFR) [12]. Osimertinib is an irreversible EGFR-TKI that selectively targets EGFR with T790M resistance mutation by forming a covalent bond with residue C797 in the ATP-binding site of mutant EGFR resistance mutations. Compared with WT EGFR, osimertinib is nearly 200 -fold more selective for mutant EGFR [13]. In several multiple multicenter Phase III clinical trials, osimertinib demonstrated its excellent efficacy and was approved by the FDA in 2015 for the treatment of patients with *EGFR* T790M-mutated NSCLC. It was initially approved as a second-line treatment for patients with T790M-acquired drug-resistant mutation NSCLC [13, 14]. Later, based on the FLAURA 3 trial, the first-line application of osimertinib in EGFR mutant NSCLC patients resulted in longer progression-free survival (PFS) and longer overall survival (OS) than the first-line application of other EGFR-TKIs [15]. In addition, osimertinib has been shown in AURA III and FLAURA trials to reduce the risk of central nervous system (CNS) metastasis in patients with T790M-mutated NSCLC compared with first- and second-generation TKIs, which is another advantage of this third-generation TKI [16]. As a result, osimertinib is approved for both first-line treatment of patients with T790M-positive and advanced *EGFR*-mutated NSCLC who have progressed on first- or second-generation EGFR-TKIs [15]. Currently, other third-generation EGFR-TKIs targeting the T790M mutation, such as Rociletinib, Olmutinib, Nazartinib, and Naquotinib are under clinical evaluation in addition to osimertinib [17, 18].

**Fig. 1:**
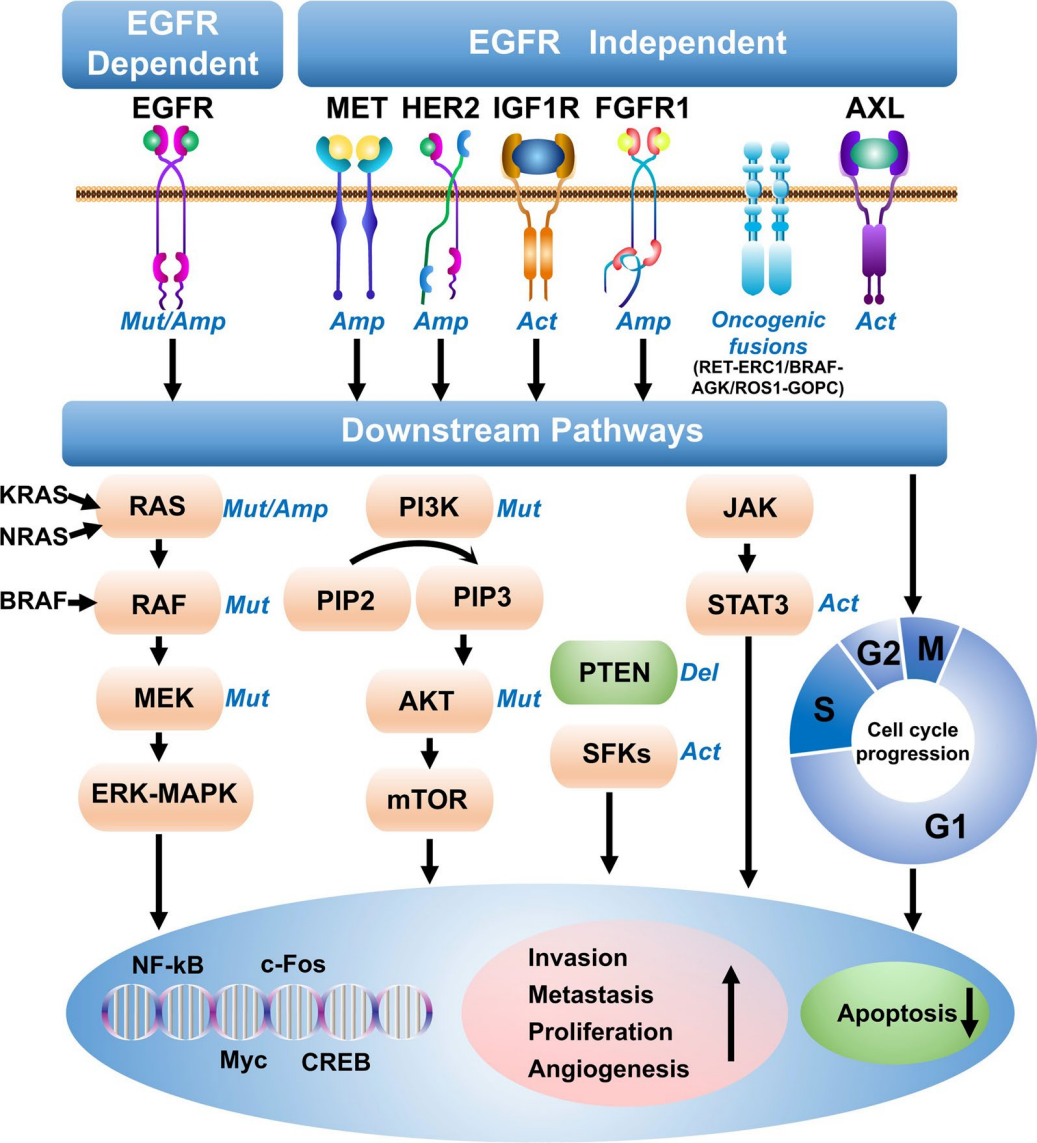
Molecular mechanisms of 3rd-generation EGFR-TKI (osimertinib) resistance, including EGFR modification (mutation/amplification), alternative pathway activation (MET/HER2/FGFR1 Amp, IGF1R/AXL Act), downstream pathway activation, epithelial-mesenchymal transition (EMT), histological transition, oncogenes fusions and cell cycle gene aberrations. Once EGFR is activated, it will cause multiple downstream signaling cascades activation such as RAS-RAF-MEK-ERK pathway, PI3K/AKT signal pathway, JAK/STAT signal transduction pathway, thus promoting transcriptional activation, cell proliferation, mitosis, anti-apoptosis, invasion and metastasis. *act* Activation; amp, amplification, *del* Deletion, *mut* Mutation

Although the efficacy of osimertinib both as the first- and second-line therapy for NSCLC is outstanding, the development of drug resistance, unfortunately, remains inevitable. In general, NSCLC patients who have been using osimertinib for 19 months in first-line therapy or 11 months in second-line therapy tend to experience relapse resistance [19–21]. A number of recent studies revealed that those resistance mechanisms are highly complex and manifold, including EGFR-dependent and EGFR-independent mechanisms (Fig. 1) [20]. In view of the current challenges of the development of the next generation of EGFR inhibitors, the mechanism of third-generation targeted drug resistances and targeted strategies are particularly important for further exploration. In this article, we review recent advancements in the types, the mechanisms and the potential therapeutic strategies for resistance to osimertinib acquired by additional *EGFR* mutations.

## EGFR and its downstream signaling pathways

**Fig. 2:**
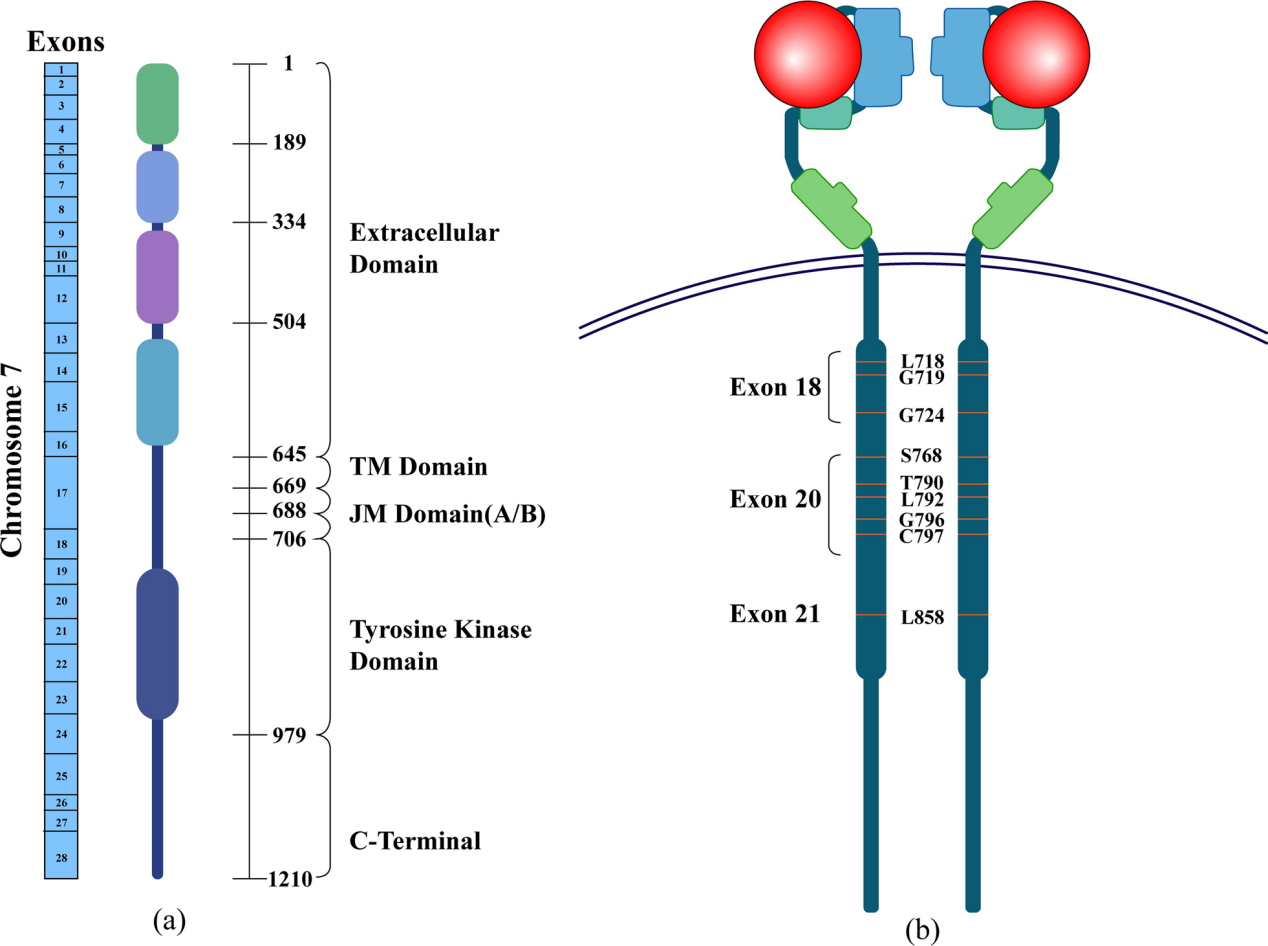
Schematic diagram of EGFR molecular domains **a** Domain of human EGFR and exons encoding it. It locates on chromosome 7, which consists of 28 exons. It consists of an extracellular domain, a hydrophobic transmembrane domain (TM domain), a juxtamembrane domain (JM domain), an intracellular protein tyrosine kinase domain that binds ligands and a C-terminal. **b** EGFR molecular structure and common EGFR-dependent drug resistance mutation sites, including L858R mutation, T790 M' gatekeeper' mutation and tertiary mutation such as C797S

The epidermal growth factor (EGF) receptor (EGFR, HER1, ErbB1) is a transmembrane receptor with tyrosine kinase activity [22]. Structurally, it consists of an extracellular domain, a hydrophobic transmembrane domain (TM domain), a juxtamembrane domain (JM domain), an intracellular protein tyrosine kinase domain that binds ligands, and a C-terminal (Fig. 2a) [23]. Its usual existent forms are monomers (inactivated) or dimers (activated), ready to bind to extracellular ligands and to activate multiple downstream cascades.

However, some mutations of *EGFR* can cause its constitutive activation even in the absence of its ligand. These are gain-of-function mutations and can be simply divided into classical activating mutations (exon 19 deletion mutation, exon 21 L858R mutation, etc.) and rare mutations [24, 25]. The exon 19 deletion and the L858R point mutation in exon 21 together account for approximately 85% of total *EGFR* mutations in NSCLC. Both of these mutations are highly correlated with the sensitivity of the first-generation EGFR-TKI, so they can be categorized as classical activating *EGFR* mutations. In comparison, rare *EGFR* mutations, including point mutations, deletions and insertions in exons 18–25 of the *EGFR* gene, have a low mutation rate, accounting for only 15% of total *EGFR* mutations [26]. The *EGFR* mutation sites are often located in the ATP-binding site of its kinase, so the consequent alteration(s) of amino acid residue(s) in this region will cause a change of the chemical properties of the binding region (such as hydrogen bond formation, hydrophobic interaction, etc.), leading to disturbance of its affinity for ATP and the stability of its inactive conformation, and finally the aberrant activation of its kinase activity. Therefore, the gain-of-function mutations of *EGFR* frequently act as carcinogenic drivers causing ligand-independent activation of downstream signaling of EGFR or sometimes drug resistance drivers, causing futility of the TKI that was supposed to be effective [25, 27].

Regardless of the way that EGFR is activated, by nature or by aberrant means, it will in turn cause multiple downstream signaling cascade activation [28]. The first downstream signaling pathway is the RAS-RAF-MEK-ERK pathway [29]. Dimerization of EGFR upon ligand binding activates RAS, resulting in the phosphorylation of MEK by RAF kinase activation. Phosphorylation of MEK sequentially triggers ERK activation, which leads to the activation of cell cycle-related transcription factors such as *C-FOS*, *MYC*, *NF-κB*, *CREB*, etc. [30]. These transcription factors further initiate the transcription of target genes, such as cyclin D, and eventually promote cell division and cell proliferation, invasion and metastasis [31]. The second signaling pathway is the PI3K/AKT signal pathway [32]. The phosphorylated EGFR tyrosine kinase acts as the binding site of PI3K to stimulate phosphatidylinositol 3, 4, 5 -triphosphate (PIP-3), which in turn activates the AKT, a master oncogenic hub molecule. Subsequently, mTOR, the key downstream regulatory molecule of AKT, is activated and starts to upregulate the expression of related proteins required for cell cycle progression from the G1 phase to the S phase and elevate the anti-apoptotic cascade signals, etc. [33]. Last but not least is the JAK/STAT signal transduction pathway, which mainly regulates immunity and mediates the proliferation and invasion of cancer cells [34, 35]. These signal transduction pathways have their respective functions and play important roles in promoting transcriptional activation, cell proliferation, mitosis, anti-apoptosis, invasion, and metastasis [36].

## EGFR mutations in exon 20

The most common *EGFR* mutation known to cause resistance to osimertinib treatment is the C797S mutation in *EGFR* exon 20 [37]. Apart from that, mutations in exon 20, including M766Q, S768I, and L718V, etc., have also been reported, and NSCLC patients with some of these mutations remain to be sensitive to afatinib [38, 39]. In addition, a series of other *EGFR* mutations located in exon 20, such as L792H/L792V and G796S/G796C mutations, are also associated with osimertinib resistance (Fig. 2b) [39].

###  Loss of T790M mutation

Loss of T790M is one consequence of the continuous treatment of third-generation EGFR-TKI and will confer resistance to osimertinib. Based on AURA3 plasma samples from 73 patients with disease progression after second-line osimertinib therapy, 36 (49%) patients were subject to loss of T790M [40]. In these 36 samples, the incidence of ex19del was higher than that of L858R (83 *vs*. 14%) [40]. This suggests that osimertinib resistance mediated by deletion of T790M is a preferential event. Another study of tumor biopsy samples from 143 osimertinib-resistant patients also showed that deletion of the T790M mutation was common (68%), and the occurrence of this deletion was associated with early resistance to osimertinib [41], further study results confirmed that T790M loss would adversely affect the PFS and OS of patients [42, 43]. It has also been suggested that the timing of the occurrence of osimertinib resistance may predict different molecular mechanisms. Early resistance is usually associated with the loss of T790M, while late resistance is associated with the retention of the T790M mutation [41].

In addition, the T790M mutation status can predict the type of acquired resistance [44]. Deletion of T790M may be misinterpreted as resensitization of tumor cells to first-line EGFR TKIs. However, T790M deletion-mediated resistance is often associated with EGFR-independent alternative or downstream competing resistance mechanisms, such as *KRAS* mutations, *MET* amplification, small cell transformation and gene fusion, etc.[39]. The occurrence of these events is not favorable for the re-implementation of EGFR-targeted therapy for first to third generations. Therefore, repeated detection of T790M mutation status after osimertinib resistance is conducive to clarifying the mechanism of osimertinib resistance and formulating the subsequent treatment strategies. Chic et al. reported on a patient with lung adenocarcinoma initially had an *EGFR* T790M mutation but progressed to *EGFR* ex19del^+^ /T790M^−^ /C797S^+^ mutation after treatment with osimertinib. At this time, the tumor is resistant to osimertinib, but the first-generation TKI gefitinib helps the patients achieve meaningful clinical improvement [45].

### C797 site mutation

The most common tertiary *EGFR* mutation is the *EGFR* C797S mutation, which occurs in exon 20 and accounts for 10–26% of cases of resistance to second-line osimertinib (the most common mechanism of resistance to second-line osimertinib), 7% of cases of resistance to first-line osimertinib treatment (the second most common resistance mechanism after *MET* amplification in first-line osimertinib resistance) [25]. Thress et al. first observed this mutation in NSCLC patients [37]. The C797S mutation refers to the substitution of cysteine by serine at codon 797 in the ATP-binding site of the EGFR tyrosine kinase domain [46, 47]. Osimertinib covalently binds to *EGFR* C797 via the stable binding of two hydrogen bonds between the pyrimidine core and M793 [48]. When C797S mutation occurs, this binding capacity is greatly reduced, thereby reducing the effect of osimertinib in inhibiting cell proliferation and EGFR phosphorylation by 100- to 1000- fold [48]. Predictably, the C797S mutation also confers cross-resistance to other irreversible third-generation TKIs by preventing their binding to the EGFR active site [49]. This suggests that these point mutations can cause steric interference, resulting in reduced affinity of these compounds to the EGFR kinase domain.

**Fig. 3:**
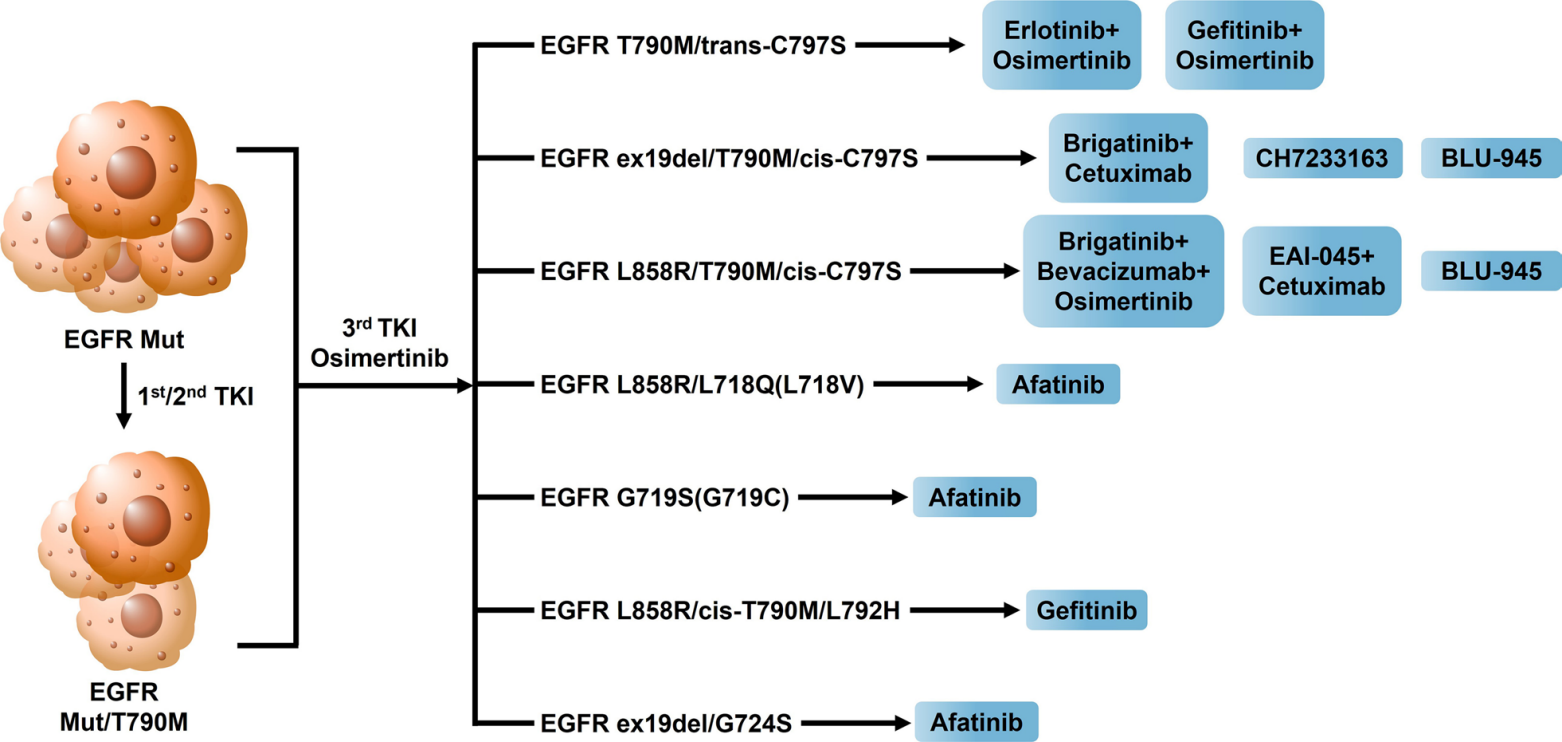
Therapeutic strategies based on clinical and vitro studies. Erlotinib combined with osimertinib, or gefitinib combined with osimertinib both have certain effects on the EGFR C797S/T790M trans configuration based on vitro studies and clinical evidence. Brigatinib combined with cetuximab or CH7233163 or BLU-945 treatment exhibited potent anti-tumor activity against the EGFR ex19del /T790M/C797S triple mutation. Brigatinib combined with bevacizumab and osimertinib or EAI045 or BLU-945 combined with cetuximab treatment, have a significant effect on the EGFR L858R-T790M-C797S triple mutation. Afatinib treatment is a potentially effective strategy for EGFR L858R/L718Q/V or EGFR G719S/C or EGFR ex19del/G724S mutation based on clinical cases or research evidence. Gefitinib can combat lung adenocarcinoma with EGFR L858R-cis T790M-L792H triple mutation in vitro

**Fig. 4:**
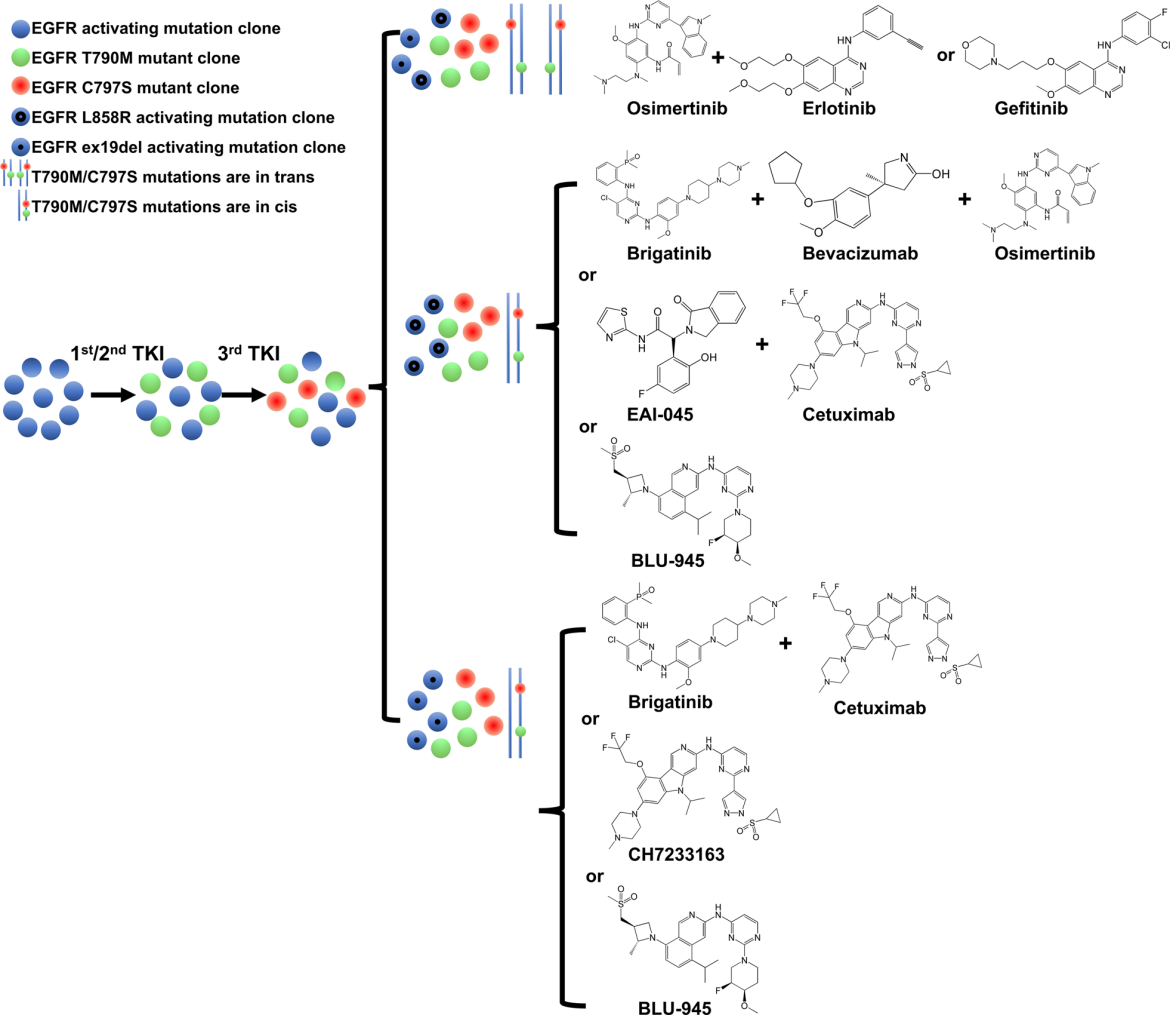
Evolutionary mechanism of drug resistance and targeted therapeutic strategies. Cell clones with primary EGFR activating mutations (e.g., L858R and ex19del mutation) appeared T790M resistant clones under the selection or induction of the 1st and 2nd generation TKIs. C797S resistant clones appeared under the same mechanism of 3rd generation TKIs. The two mutated genes, C797S and T790M, can be located on the same DNA strand (in cis) or on different DNA strands (in trans). There are now different effective therapeutic strategies for these specific combinations of drug-resistant mutations that have evolved from drug selection

Importantly, the allelic information for C797S has potential therapeutic implications. According to in vitro studies, the C797S mutation and T790M mutation occur in cis (on the same allele) or trans (on a different allele). The C797S/T790M trans configuration accounts for 15% of the mutations, and cells with this configuration are sensitive to the combined treatment of first- and third-generation EGFR-TKIs [25, 50]. Wang and colleagues reported the first clinical evidence of the efficacy of the first- and third-generation EGFR-TKIs in combination with targeted therapy against the *EGFR* C797S/T790M trans configuration [50, 51], and found that erlotinib combined with osimertinib, or gefitinib combined with osimertinib both have certain effects (Figs. 3–4). However, the C797S/T790M cis configuration, which accounts for 85% of *EGFR* mutations, is resistant to osimertinib alone and in combination treatment [51].

At the same time, C797 also has a rare mutation form: C797G. Yang et al. studied the plasma samples from 93 advanced NSCLC patients treated with osimertinib as second-line therapy and found that 1/3 of the patients developed *EGFR* tertiary mutation (such as *EGFR*m/T790M/C797S) after osimertinib resistance [52]. Among the patients with *EGFR* tertiary mutations, 24% were C797S mutations, and C797G/C797S mutations were found to coexist in 2 cases. Next-generation sequencing of pleural biopsy specimens from one of these patients was accompanied by *MYC* and EGFR amplification [53].

### G796 site mutation

G796 is adjacent to residue C797, located below the phenyl aromatic ring of osimertinib, and occupies the solvent-front position, which is the area on the surface of the kinase where many inhibitors are attached, and the use of kinase inhibitors is often easy to trigger the mutations here in turn leading to drug resistance. For example, TKIs of *ALK*, *ROS1*, and *RET* often cause mutations in the solvent-front position. G796 mutant forms are G796R, G796S, and G796D, and the substituted residues may interfere with osimertinib in the solvent-front aromatic ring [54]. It is worth noting that the co-crystal structure of osimertinib and EGFR shows that the "hydrophobic interlayer" formed between L718 and G796 is exactly the site of the hydrophobic interaction of aromatic rings in the solvent-front of osimertinib. Both mutations disrupt hydrophobic interactions and interfere with osimertinib binding to the kinase domain. G796R interferes with osimertinib-EGFR binding more strongly than G796S [54]. In addition, *EGFR* G796D mutation was also detected in second-line osimertinib-resistant patients, and neither the first-generation TKI nor osimertinib could inhibit *EGFR* G796D-driven cell proliferation [52, 55]. Although there are no targeted therapy options, in vitro studies have shown that tumor cells with G796X mutations proliferate much slower than L792X and T790M mutant cells, and patients with these mutations may be more suitable for conventional chemotherapy. However, Lin's team has now established a computer model study of 1058 osimertinib-resistant patient samples and found that the first-generation TKI gefitinib still has the activity of binding to EGFR in the presence of the G796S mutation, which may be a way to overcome the G796S mutation. One of the potential therapeutic strategies for drug resistance mutations [56].

### L792 site mutation

The N-lobe and C-lobe of the tyrosine kinase domain in the cytoplasm of the EGFR molecule are connected by the so-called "hinge" region of the kinase [57]. Computer simulations suggest that mutations of residue L792 in this region can sterically interfere with the methoxy group on the phenyl ring of osimertinib and bend its connection to the kinase domain, disrupting the positioning and combination of osimertinib and the ATP-binding pocket [58]. L792H is the most common hinge region mutation in the L792 residue. L792 mutation usually coexists with other *EGFR* mutations, such as T790M can coexist in cis form, and G796/C797 can coexist in trans form [58, 59], which indicates that L792 mutation may independently lead to osimertinib resistance. Furthermore, the L792 mutant in vitro remained sensitive to the first-generation TKI gefitinib (Fig. 3) [60].

### Rare mutation in exon 20

In addition to the well-known *EGFR* tertiary mutations that lead to osimertinib resistance, other mutations in exon 20 rarely occur after osimertinib resistance. *EGFR* S768I is a rare mutation in exon 20 that has been detected in patients who progressed on second- and first-line osimertinib therapy, often co-occurring with sensitizing *EGFR* mutations [39, 61], but the exact mechanism and prognostic impact have not been fully elucidated. What is currently known is that S768I, as part of the αC helix in the kinase domain of EGFR, stabilizes the active 'αC-in' conformation by improving the hydrophobic accumulation between the αC-helix and the adjacent β9 strand, reducing osimertinib sensitivity [62]. One patient also reported an exon 20 insertion (1%) in second-line osimertinib failure cases [39, 63].

## EGFR mutations in exon 18

### L718 and G719 site mutation

In vitro and in vivo studies have shown that mutations in the L718 residue can also lead to osimertinib resistance. The L718 residue is located near the highly conserved flexible glycine loop in the N-Lobe of the EGFR kinase domain, which is the binding site for ATP [63]. The common drug-resistant mutations generated on L718 are L718Q and L718V, of which the former has a higher frequency and drug resistance. Computer simulations showed that the L718Q mutation causes steric restriction and reduces the hydrophobic interaction of osimertinib with EGFR, thereby hindering the binding to EGFR [64]. The first evidence of L718Q mutation was found in an *EGFR* L858R/T790M co-mutated metastatic NSCLC patient treated with osimertinib [64]. However, in vitro studies have shown that *EGFR* ex19del/T790M/L718Q co-mutated cells remained sensitive to osimertinib. So it is speculated that L718Q-mediated resistance preferentially occurs at L858R rather than ex19del [64]. Notably, *EGFR* L718Q was also found to be a resistance mechanism to first-line osimertinib therapy (2%) [39]. Meanwhile, according to Yang et al., most patients with L718Q/V mutation did not have coexisting C797 mutation (6/7), suggesting that mutation at L718 may be another strong candidate factor mediating osimertinib resistance, which is mutually exclusive with the C797 mutation [52].

In addition, the G719X mutation in *EGFR* exon 18 is one of the more common rare mutations in *EGFR*, including G719A, G719D, and G719C [44, 52]. On the one hand, since the L718 residue is very close to the G719A residue, similar to the mechanism of L718, the spatial limitation predicted by computer simulation may also be the reason for the resistance of G719A mutation patients to osimertinib [52]. On the other hand, The *EGFR* G719X mutation can lead to constitutive activation of the EGFR [65].

Based on current studies, the L718Q/V mutation occurs almost exclusively in the context of the L858R driver mutation, and both in vitro and in vivo models have shown sensitivity to afatinib, as well as resistance to erlotinib and osimertinib [66]. Clinically, Liu et al. reported a patient with *EGFR* L858R/L718Q-mutated NSCLC. After treatment with afatinib, the patient's clinical symptoms were relieved, and the ECOG score was significantly improved [67]. Song also reported a patient with L718V mutation persistently sensitive to afatinib therapy [68]. All the above evidence suggests that afatinib treatment is a potentially effective strategy for L718Q/V mutation (Fig. 3).

For G719X mutations, current studies have found that different types of G719X mutations may retain sensitivity to different types of TKIs, depending on the different substitution types of amino acid residues [47]. In vitro studies, Shinichi Kimura's team found that G719S mutant cells were sensitive to afatinib [69], and similarly, G719C mutants were also sensitive to afatinib (Fig. 3) [70]. However, a small number of G719X mutations are still sensitive to osimertinib. In a case of a patient with L747S/G719C mutation reported by Emmanuel Grolleau et al., the tumor shrank significantly after treatment with osimertinib, showing that the L747S/G719C mutation is sensitive to osimertinib [71].

### G724 site mutation

Researchers have identified the G724S mutation in the ATP phosphate-binding loop (P-loop) of the EGFR tyrosine kinase domain in multiple osimertinib-resistant patients [72]. Structural analysis shows that the G724S mutation affects the adjacent ELREA sequence (the ATP-binding site of the EGFR-TK region), modulates the αC helix structure in the kinase domain, and then affects the structure and dynamics of the binding site, interfering with osimertinib binds to EGFR [73]. The researchers speculate that G724S-mediated resistance preferentially occurs at ex19del rather than the L858 locus, that is, G724S mutation is a specific resistance mechanism to osimertinib ex19del, and its occurrence is highly correlated with ex19del [73]. Interestingly, the second-generation TKI still retains the kinase affinity in the G724S mutant. Benjamin's team simulated the stable binding of afatinib to the *EGFR* G724S mutant by computer modeling and verified in vitro that afatinib could effectively overcome G724S-driven drug resistance [73]. At the same time, Fang's clinical case reported that in a patient with osimertinib-resistant *EGFR* G724S/ex19del lung adenocarcinoma, afatinib treatment achieved significant results, indicating that G724S mutant tumors may still be sensitive to afatinib in vivo, which provides a promising therapeutic strategy for patients with such mutations(Fig. 3) [74].

## EGFR copy number alterations

EGFR amplification and copy number alterations are also important resistance mechanisms. It translates to the overexpression of EGFR protein, which in turn leads to aberrant activation of several downstream signal regulation pathways such as RAS/RAF/MAPK and PI3K/AKT/mTOR and STAT signaling pathway etc., and consequently promotes tumor occurrence and progression even in the presence of EGFR TKI. Moreover, EGFR amplification often occurs concurrently with EGFR*-*TKI mutations. Therefore, it is difficult to determine whether EGFR amplification is a primary or acquired mechanism of osimertinib resistance [75, 76]. Roper et al. found that in the presence of osimertinib resistance, the mutated *EGFR* allele (rather than the wild-type allele) was further amplified. This suggests that further amplification of the *EGFR* mutant allele is more common in acquired resistance to osimertinib [77]. Franciele H. Knebel detected significant and unequal amplification of *EGFR* ex19del copy number in the blood of a patient with *EGFR* ex19del/T790M/C797S mutated osimertinib-resistant NSCLC, suggesting that selective amplification of the *EGFR*-ex19del allele may represent a novel resistance mechanism to osimertinib [76]. A cohort analysis by Le et al. showed that EGFR amplification occurred in 19% (8/42) of 42 osimertinib-resistant NSCLC patients [78]. In another study, Helman et al. found that 29% (17/58) of osimertinib-resistant patients developed EGFR amplification [79].

## The evolution of osimertinib-resistant subclone

Subclones of various mutation types exist in advanced NSCLCs. Under the strong selection pressure of EGFR-TKI, tumor cells undergo clonal selection. Clones with primary or secondary drug-resistant mutations survive and replicate for many cycles, replacing those drug-sensitive clones and ultimately driving the progression of drug resistance and tumor relapse [79]. The T790M mutation that leads to the acquisition of resistance of EGFR to the first and second-generation TKIs is such a winner in the subclonal selection mechanism of resistance. Alternatively, Hata et al. found that drug-resistant clones can pre-exist or evolve from earlier drug-resistant cells, so those survived the initial treatment can act as evolutionary ancestors for subsequent drug-resistant cells [80]. Similarly, although the current study has not yet determined whether or not the EGFR-dependent resistance mutations of osimertinib led by C797S are derived from pre-existing subclones, we can reasonably speculate that it has a similar development trajectory as above, and conduct exploratory research based on this idea, and ultimately elucidate the resistance mechanism.

While resistance originated from evolutionary pressures to select specific subclones, resistance to TKIs is often associated with the emergence of novel mutations in the resistance-related genes or in the targeted gene itself. For example, a recent study revealed that the treatment response to osimertinib is often incomplete due to the additional co-occurring genetic alterations together with EGFR mutations. As an example, they reported the loss of function mutations of RBM10 that co-occur with mutant EGFR decreased the efficacy of osimertinib in patient-derived EGFR-mutant tumor models. The reason, as they inferred, was the inactivation of RBM10 decreased the ratio of (proapoptotic) Bcl-xS to (antiapoptotic) Bcl-xL isoforms of Bcl-x and therefore diminished EGFR inhibitor–mediated apoptosis [81]. Interestingly, the origin of such recurrent mutations can also be attributed to the strong apoptosis inductive effect of TKIs, and finally resulted in drug-induced mutation [82]. TKI induces repeated apoptosis which in turn triggers the down-regulation of genes involved in mismatch repair and homologous recombination DNA repair. Consequently, these events lead to the increased error rate of DNA polymerase, making damage repair more prone to errors and thus increasing the incidence of *EGFR* mutations [83]. For example, Russo et al. found that drug-resistant cancer cells that survived EGFR inhibition exhibited reduced expression of mismatch repair (MMR) genes *MLH1* and decreased expression of *MSH2*, *MSH6*, and homologous recombination (HR) repair effectors *BRCA2* and *RAD51*, thus shifting DNA polymerase from high fidelity to low fidelity, leading to damage repair system more error-prone and transiently increases its mutagenic capacity [83]. The above additional *EGFR* mutations are associated with the disruption of osimertinib binding to tyrosine kinase by altering the binding site through allosteric/conformational transitions and altering the target through EGFR-TKI susceptibility mutations (specifically the C797S mutation).

## Development of the fourth-generation TKI

Since the C797S mutation is the most abundant cis configuration (85%), overcoming the C797S-mediated resistance to the third-generation TKIs has become a hot issue for both researchers and pharmaceutical companies. As shown in Table 1, two potential solutions to solve this problem is the combination therapy and the development of the fourth-generation TKI [10]. Especially for the latter, it started coming into public attention as all the currently developed fourth-generation TKIs, such as brigatinib, EAI045, CH 7,233,163, etc., exhibited good curative effects in NSCLC patients with C797S mutation [84–86]. For example, brigatinib (Alunbrig), a novel dual kinase inhibitor of ALK and EGFR, was approved by FDA in April 2017 and used for first-line treatment of locally advanced or metastatic ALK-positive NSCLC patients [87]. It can effectively suppress the proliferation of ex19del-T790M-C797S triple mutant cells in vitro and in vivo, and it was found to be more effective when combined with the anti-EGFR antibody cetuximab [48]. Meanwhile, Wang et al. reported that a patient with *EGFR* ex19del-mutated lung adenocarcinoma was initially treated with gefitinib and then osimertinib, followed by disease progression after gaining triple mutation of *EGFR* ex19del/T790M/cis-C797S, and achieved significant efficacy by using brigatinib in combination with cetuximab (Figs. 3–4) [88]. A retrospective analysis of 15 patients with osimertinib resistance who also carried C797S/T790M mutations showed that the Objective Response Rate (ORR) reached 60% after using brigatinib combined with cetuximab, and the Disease Control Rate (DCR) reached 100% [89]. In addition to anti-EGFR antibodies, brigatinib can also be combined with the vascular endothelial growth factor (VEGF) inhibitor bevacizumab, in combination with osimertinib to effectively combat lung adenocarcinoma with *EGFR* L858R-T790M-cis C797S triple mutation (Figs. 3–4)[90].

**Table 1:** The next generation TKIs/TKIs combination currently undergoing development and clinical trials

The fourth – generation TKI	Suppression	Study	Outcome	References
Brigatinib	EGFR ex19del/T790M/C797S Mutation	In vitro and in vivo research	Effective	Uchibori K et al. 2017 [48]
Brigatinib + Cetuximab	EGFR ex19del/T790M/cis-C797S Mutation	A retrospective analysis of 15 patients	ORR 60%; DCR 100%	Wang Y et al., 2020 [89]
Brigatinib + Cetuximab + Osimertinib	EGFR L858R/T790M/cis-C797S Mutation	A case report	Partial remission was observed after one month of the treatment	Zhao J et al., 2018 [90]
EAI045 + Cetuxi mab	EGFR ex19del/T790M/C797S Mutation	In vitro and in vivo research	Effective	Jia Y et al., 2016 [91]
CH7233163	EGFR ex19del/T790M/C797S Mutation	In vivo resarch	Effective	Kashima K et al., 2020 [92]
BLU-945	EGFR + /T790M;EGFR + /T790M/C797S Mutation	In a phase ½ clinical trial (NCT 04862780)	In progress	Eno MS et al., 2022 [93]

*TKI* Tyrosine Kinase Inhibitor

In addition to brigatinib, Yong Jia's team found that the combination of the novel EGFR allosteric inhibitor EAI045, combined with cetuximab, has a significant effect on the L858R-T790M-C797S triple mutant but not on the ex19del mutant NSCLC cells (Figs. 3–4) [91]. Fortunately, another fourth-generation TKI, CH7233163, exhibited even more potent anti-tumor activity against the *EGFR* ex19del-T790M-C797S triple mutation (Figs. 3–4) [92]. Even though so far an ideal fourth-generation TKI is yet to come, the future is sure to be optimistic as more and more candidates with better therapeutic effects and pharmacokinetic and pharmacodynamic properties are emerging.

## Conclusion and future perspectives

The mechanisms of multiple EGFR-dependent osimertinib resistance have been extensively studied from the bench to the bed side, but a comprehensive and standardized solution to each type of the osimertinib resistance is so far not available. Considering the central role of osimertinib in the treatment of *EGFR*-mutanted NSCLC, it is urgent to elucidate various types of drug resistance mechanisms and develop corresponding strategies. Importantly, next-generation sequencing is nowadays widely applied in detecting the plasma ctDNA for tumor re-biopsy. It not only tells the tumor genotypes, together with other clinical data and omic analysis provide very useful information for understanding specific osimertinib resistance mechanisms in a clinical setting and decision-making for the follow-up treatment strategy.

Chemotherapy remains as an indispensable solution until newer generations of targeted drugs are approved. At the same time, ICIs, such as PD-1 and PD-L1, have made breakthroughs in the immunotherapy of advanced NSCLC [94]. Despite that large-scale trials showed negative outcomes in the treatment of *EGFR* mutation-driven NSCLC, there are also a number of studies reporting positive results from the combined treatment of ICIs and chemotherapy or the combination of ICIs and EGFR-TKIs for the treatment of *EGFR*-mutated NSCLC [7, 95–98]. In addition, chimeric antigen receptor T cell (CAR-T) immunotherapy is also a new idea for the treatment of NSCLC [99, 100], but its efficacy in patients with *EGFR* mutations still needs support from more research and case studies.

Finally, large-scale clinical trials like the ORCHARD trial need to be conducted in the osimertinib-resistant cohort in addition to preclinical in vitro and in vivo studies to evaluate the effectiveness of various treatment options. Excitingly, the development and early trials of fourth-generation EGFR-TKIs targeting tertiary *EGFR* mutations have made great progress. It is foreseeable that with the continuous progress of next-generation EGFR inhibitors, chemotherapy, antibodies, and immune checkpoint inhibitors, there will be more and better solutions in the toolbox of clinicians when they confront the osimertinib-resistant NSCLC in the future.

## Data Availability

The original contributions presented in the study are included in the article, further inquiries can be directed to the corresponding authors.

## Abbreviations

EGFR: Epidermal growth factor receptor
TKI: Tyrosine kinase inhibitor
EGFR-TKI: Epidermal growth factor receptor tyrosine kinase inhibitor
NSCLC: Non-small cell lung cancer
PFS: Progression free survival
ATP: Adenosine triphosphate
WT: Wild type
ErbB: Erythroblastic oncogene B
RAS: Rat sarcoma
RAF: Rapidly accelerated fibrosarcoma
MEK: METhyl ethyl ketone
MAPK: Mitogen-activated protein kinase
ERK: Extracellular regulated protein kinase
FOS: Finkel-Biskis-Jinkins murine osteogenicsarcoma
MYC: Myelocytomatosis
NF-κB: Nuclear factor kappa-B
CREB: CAMP-response element binding protein
PI3K: Phosphatidylinositide 3-kinases
AKT: (PKB: protein kinase B)
PIP-3: Phosphatidylinositol-3, 4, 5-phosphate
mTOR: Mechanistic target of rapaMYCin
JAK: Janus kinase
STAT: Signal transducer and activator of transcription
TK: Tyrosine kinase
mPFS: Median PFS
CNS: Central nervous system
MET: Mesenchymal-epithelial transition factor
Ex19del: Exon 19 deletion
OS: Overall survival
NGS: Next generation sequencing
KRAS: Kirsten rat sarcoma viral oncogene homolog
BRAF: V-RAF murine sarcoma viral oncogene homolog B1
ctDNA: Circulating tumor DNA
